# Synephrine Inhibits Eotaxin-1 Expression via the STAT6 Signaling Pathway

**DOI:** 10.3390/molecules190811883

**Published:** 2014-08-08

**Authors:** Kyung-Baeg Roh, Il-Hyun Kim, Young-Soo Kim, Myungjae Lee, Jung-A Lee, Eunsun Jung, Deokhoon Park

**Affiliations:** Biospectrum Life Science Institute, Sangdaewon-Dong, Seongnam-City, Gyeonggi-Do 442-13, Korea; E-Mails: biosh@biospectrum.com (K.-B.R.); biotc@biospectrum.com (I.-H.K.); biosf@biospectrum.com (Y.-S.K.); biocf@biospectrum.com (M.L.); biofk@biospectrum.com (J.-A.L.)

**Keywords:** *Citrus unshiu*, synephrine, STAT6, eotaxin-1, eosinophils

## Abstract

*Citrus* contain various flavonoids and alkaloids that have multiple biological activities. It is known that the immature *Citrus* contains larger amounts of bioactive components, than do the mature plants. Although *Citrus* flavonoids are well known for their biological activities, *Citrus* alkaloids have not previously been assessed. In this study, we identified synephrine alkaloids as an active compound from immature *Citrus unshiu*, and investigated the effect of synephrine on eotaxin-1 expression. Eotaxin-1 is a potent chemoattractant for eosinophils, and a critical mediator, during the development of eosinophilic inflammation. We found that synephrine significantly inhibited IL-4-induced eotaxin-1 expression. This synephrine effect was mediated through the inhibition of STAT6 phosphorylation in JAK/STAT signaling. We also found that eosinophil recruitment induced by eotaxin-1 overexpression was inhibited by synephrine. Taken together, these findings indicate that inhibiting IL-4-induced eotaxin-1 expression by synephrine occurs primarily through the suppression of eosinophil recruitment, which is mediated by inhibiting STAT6 phosphorylation.

## 1. Introduction

Synephrine is an alkaloid that was initially isolated as a synthetic organic compound, first isolated as a natural product from the leaves of various *Citrus* trees, and which presence has been noted in different *Citrus* juices [1,2]. The peel of unripe *Citrus* fruits is the part of the plant that has the highest level of synephrine [3]. The concentration of synephrine is higher in fruits of small diameter, when compared to larger ones, probably due to the higher levels found in their peels, which decrease with the maturation of the fruit [4,5]. Synephrine is also found in the human organism, where it is considered a trace amine, due to its low plasmatic levels [6]. The preparations used in Traditional Chinese Medicine (TCM) are the immature and dried whole fruits from *Citrus aurantium*[7]. Extracts of *Citrus* fruits or purified synephrine are also marketed in the US, sometimes in combination with caffeine, as a weight-loss-promoting dietary supplement for oral consumption. Chemically, synephrine is similar to adrenaline or epinephrine. Synephrine activates several types of adrenergic receptors [8,9]. Therefore, it is used pharmacologically as a sympathomimetic agent, with vasoconstrictor and bronchiectatic agent.

Eosinophils are crucial effector cells, in the inflammatory reactions associated with allergic-inflammatory diseases and parasitic infections. Their products, such as cytotoxic granule proteins, leukotrienes, and cytokines, are involved in the pathological changes seen at the sites of inflammation. Eosinophils are recruited to the sites of inflammation by locally released chemotactic agents. The CC chemokine, eotaxin-1, a potent chemoattractnat for eosinohpils, is a critical mediator for the development and perpetuation of allergen-induced eosinophilic inflammation [10]. Eotaxin-1 is produced at high levels in patients with atopic dermatitis, and is localized to the inflammation site. This concentrated expression may preferentially target eosinophils to the epithelium, and induce degranulation, leading to the release of epithelium-damaging proteins [11].

The JAK-STAT signaling pathway becomes activated, in response to cytokines like interleukins (IL), interferons (IFN), and certain peptide hormones [12,13,14], all of which are thought to have biologically significant roles in inflammatory diseases [15]. IL-4 is a pivotal cytokine associated with allergic disease that supports Th2 development, and that is produced by Th2 cells, basophils, and mast cells [16,17,18]. The transcription factor STAT6 was shown to be responsible for the IL-4-mediated induction of eotaxin-1 [19], and could contribute to allergen-induced airway eosinophilia, and eosinophilic inflammation [20,21]. Therefore, investigating the possible involvement of various JAK-STAT regulators in immune disease and the associated molecular mechanisms is an important area of research.

In the present study, we investigated the effects of synephrine on IL-4-induced eotaxin-1 production, and its mechanisms of action. In addition, we demonstrated that synephrine inhibits STAT6 phosphorylation, during IL-4-induced upregulation of eotaxin-1 in fibroblasts.

## 2. Results and Discussion

### 2.1. Immature Citrus unshiu Fruit Extracts Inhibit Eotaxin-1 Expression Induced by IL-4

**Figure 1:**
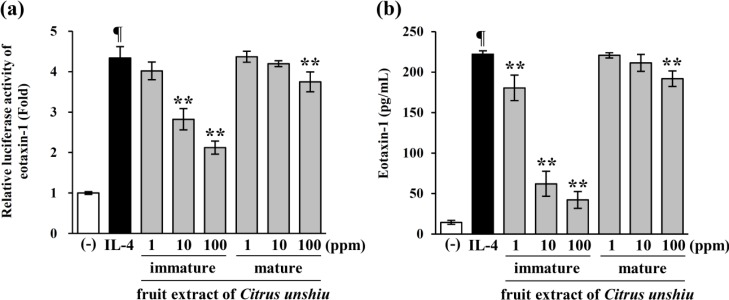
Immature *Citrus unshiu* fruit extracts inhibit eotaxin-1 expression. (**a**) The eotaxin-1 gene promoter-luciferase reporter vector was transfected into 70% confluent NIH/3T3 cells, and cultured for 24 h. The cells were pretreated with immature and mature *Citrus unshiu* fruit extracts at 1, 10, 100 ppm, respectively, for 1 h, and then stimulated with IL-4. Luciferase activity was calculated, against IL-4-unstimulated control; (**b**) Cells were pretreated with immature and mature *Citrus unshiu* fruit extract at 1, 10, 100 ppm, respectively, for 1 h, and then further incubated with IL-4 (50 ng/mL), for 24 h. Eotaxin-1 release was then determined, using an ELISA. Data are representative of at least three independent experiments. Results are mean ± standard deviation (SD) (*n* = 3). ¶ *p* < 0.05 *vs.* IL-4-untreated control. ** *p* < 0.05 *vs.* IL-4-treated control.

A previous study suggested that immature *Citrus unshiu* extract showed anti-allergic effect [22]. We first investigated the effects of *Citrus unshiu* fruit extract on IL-4-induced expression of eotaxin-1 in NIH/3T3 cells. A luciferase reporter assay and an ELISA were performed, to measure eotaxin-1 expression. As shown in Figure 1a, IL-4-induced activation of eotaxin-1 promoter was significantly inhibited by immature *Citrus unshiu* fruit extracts; whereas, mature *Citrus unshiu* fruit extracts slightly inhibited the expression of eotaxin-1 induced by IL-4. Eotaxin-1 protein levels were also inhibited by immature *Citrus unshiu* fruit extracts; however, mature *Citrus unshiu* showed weak effects on eotaxin-1 expression induced by IL-4 (Figure 1b). These results indicated that immature *Citrus unshiu* contain more active ingredients for inhibiting eotaxin-1 expression induced by IL-4, than do mature *Citrus unshiu*.

### 2.2. Immature Citrus unshiu Extracts Contained more Phytochemical Contents than Mature Citrus unshiu Extracts

**Figure 2:**
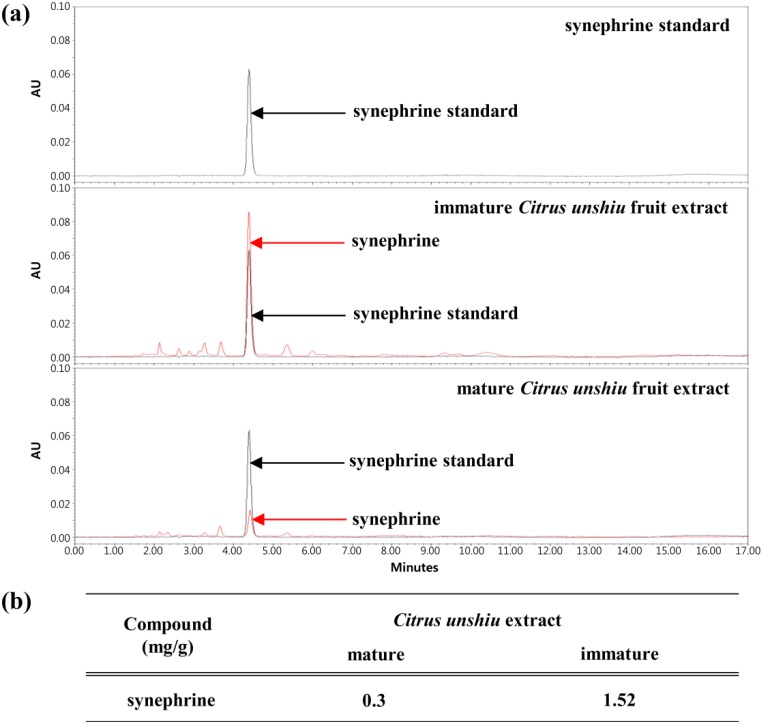
Phytochemical analysis between immature *Citrus unshiu* and mature *Citrus unshiu* fruit extract. (**a**) Contents of synephrine were analyzed by high performance liquid chromatography (HPLC). (**b**) Quantitative analysis of synephrine in mature *Citrus unshiu* fruits and immature *Citrus unshiu* fruits extracts.

To identify the active compounds in immature *Citrus unshiu*, we analyzed the differences of phytochemicals between mature and immature *Citrus unshiu* fruit extracts. Hesperidin and naringin were mainly contained in *Citrus unshiu* extract, at higher contents than other flavonoids; and the content of these flavonoids decreased during maturation. These flavonoids are known for their anti-inflammatory effect [23]. Synephrine alkaloid was found in *Citrus unshiu* fruit extract; and immature extract contained approximately five times more synephrine than mature extract (Figure 2b). As shown in Figure 2a, the greatest difference between the two types of extracts was the content of synephrine, which is the main alkaloid in *Citrus* fruits.

### 2.3. Synephrine Suppresses IL-4-induced Eotaxin-1 Production in NIH/3T3

**Figure 3:**
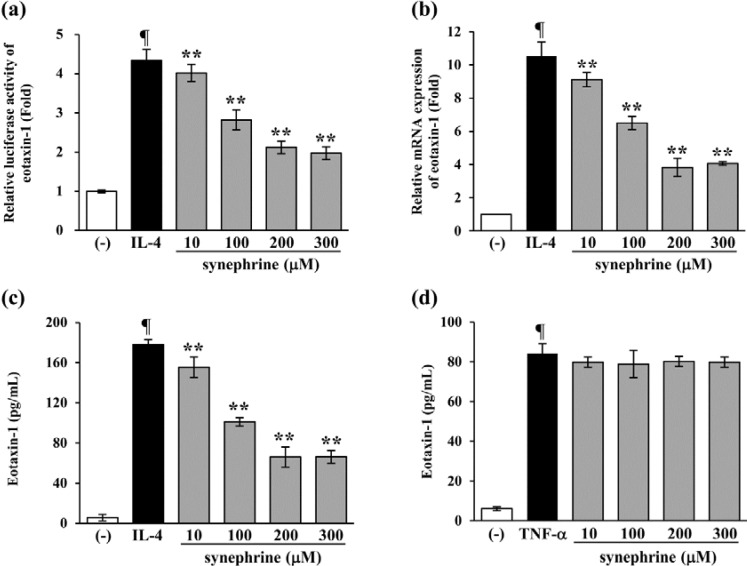
Effect of synephrine on eotaxin-1 gene expression. (**a**) The eotaxin-1 gene promoter-luciferase reporter vector was transfected into 70% confluent NIH/3T3 cells, and cultured for 24 h. The cells were pretreated with synephrine for 1 h, and then stimulated with IL-4. Luciferase activity was calculated against IL-4-unstimulated control; (**b**) Cells were cultured in serum-free DMEM, in the presence of indicated concentrations of synephrine, for 24 h. Total RNA was isolated, and eotaxin-1 mRNA levels were assayed by quantitative real-time PCR. Data are representative of at least three independent experiments; (**c**) Cells were pretreated with synephrine for 1 h, and then further incubated with IL-4 (50 ng/mL), for 24 h. Eotaxin-1 release was then determined, using an ELISA. (**d**) Cells were pretreated with the indicated concentrations of synephrine for 1 h, and then further incubated with TNF-α (50 ng/mL), for 24 h. Eotaxin-1 release was then determined, using an ELISA. Results are mean ± standard deviation (SD) (*n* = 3). ¶ *p* < 0.05 *vs.* IL-4-untreated control. ** *p* < 0.05 *vs.* IL-4-treated control.

Synephrine exist in two different positional isomeric forms (meta, *m*- and para, *p*-) in natural sources. These two isomers of synephrine do not have equivalent properties. To investigate the effect of synephrine on eotaxin-1 expression, we added various concentrations of synephrine (10 μM, 100 μM, 200 μM, and 300 μM) to fibroblasts, NIH/3T3, 1 h before stimulation for 24 h with IL-4 (50 ng/mL). IL-4-induced eotaxin-1 production was significantly inhibited by *p*-synephrine; whereas, *m*-synephrine slightly inhibited the production of eotaxin-1 induced by IL-4 (data not shown). Therefore, *p*-synephrine was used, for further experiments. As shown in Figure 3a, IL-4-induced activation of the eotaxin-1 promoter was attenuated by synephrine. In addition, synephrine reduced eotaxin-1 mRNA levels, in a concentration-dependent manner (Figure 3b). Consistent with these results, eotaxin-1 protein levels were also inhibited by synephrine (Figure 3c). However, synephrine showed no effects on eotaxin-1 expression induced by TNF-α, one of the main cytokines involved in eotaxin-1 expression (Figure 3d). These results indicate that synephrine exerts an inhibitory effect on IL-4-induced eotaxin-1 expression, and suggest that its effect is specific to IL-4-induced signaling.

### 2.4. Synephrine Inhibits STAT6, but not JAK1 Phosphorylation Induced by IL-4

**Figure 4:**
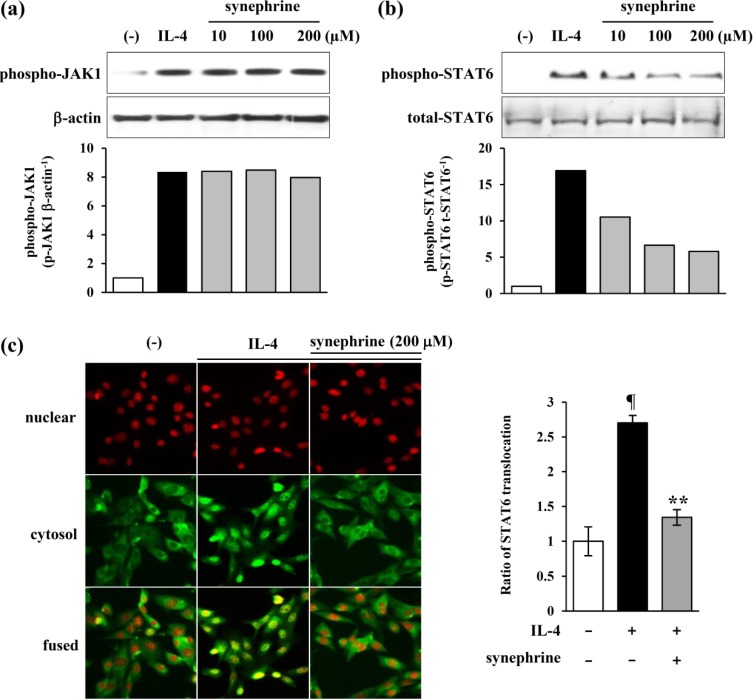
Effects of synephrine on the activation of JAK1 and STAT6. NIH/3T3 cells were starved for 24 h, and then treated with IL-4 (50 ng/mL), in the presence of synephrine. Cell lysates were prepared at 10 min, and then subjected to Western blot analysis. The bands for phospho-JAK1 were detected, and normalized to that of β-actin (**a**); and STAT6 bands were detected, and normalized to that of total STAT6 (**b**). (**c**, **d**) NIH/3T3 cells were treated with synephrine for 3 h, before stimulation with IL-4 (50 ng/mL). After 1 h stimulation, the cells were fixed, permeabilized, and then incubated with rabbit antibodies against STAT6, for 2 h. Thereafter, cells were stained with FITC-conjugated anti-rabbit IgG, and nuclei were stained with Hoechst 33342. Translocation rate was calculated, using the IN Cell Analyzer 1000 instrument. Data are representative of at least three independent experiments. ¶ *p* < 0.05 *vs.* IL-4-α-untreated control. ** *p* < 0.05 *vs.* IL-4-treated control.

STAT6 acts as a signal transducer immediately downstream of IL-4 receptor activation, via tyrosine phosphorylation by JAK1, IL-4 receptor-associated tyrosine kinases [24]. In the previous studies, it was reported that STAT6 was essential for IL-4-induced eotaxin-1 production [25]. To investigate whether these signaling factors were involved in the synephrine effects, we performed Western blot analysis for JAK1 and STAT6. As shown in Figure 4, STAT6 and JAK1 phosphorylation were induced by IL-4. In addition, STAT6 phosphorylation was inhibited by synephrine (Figure 4b), whereas synephrine had no effect on JAK1 phosphorylation (Figure 4a). These results suggest that synephrine attenuates IL-4-induced eotaxin-1 expression, by downregulating the activation of STAT6. We performed immunohistochemistry for STAT6, to analyze STAT6 translocation from the cytosol to nuclei. The immunohistochemical studies revealed that the STAT6 translocation rate decreased by synephrine treatment (Figure 4c). These results indicate that synephrine is involved in attenuating IL-4-induced-STAT6 translocation.

### 2.5. IL-4 Induced Eotaxin-1 Expression is Mediated by JAK/STAT Signaling

**Figure 5:**
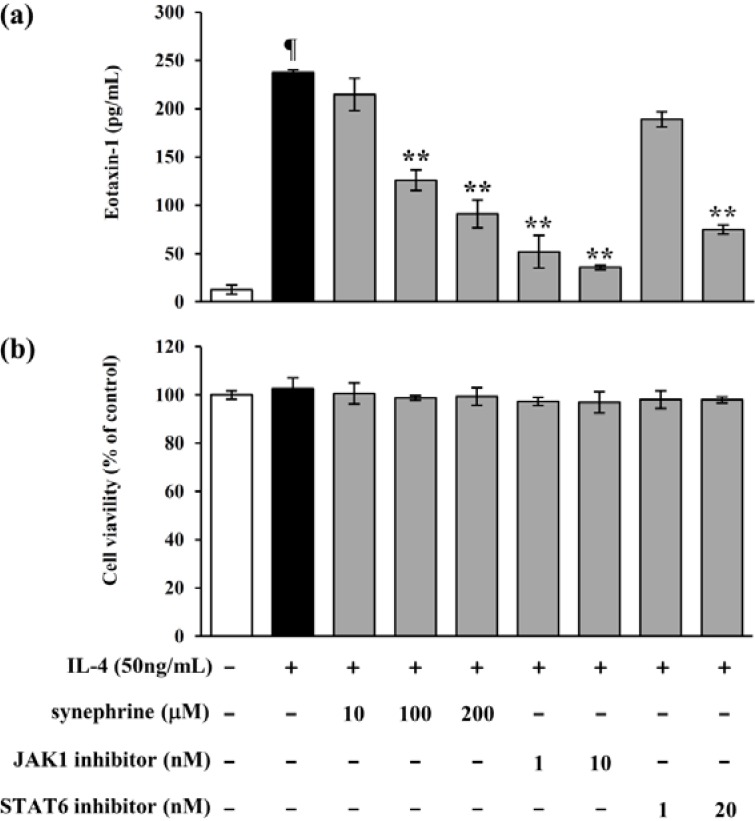
Effects of signal inhibitors on eotaxin-1 expression in NIH/3T3 cells. (**a**) NIH/3T3 cells were pretreated with JAK1 inhibitor, pyridone 6 or STAT6 inhibitor, AS1517499 for 3 h, and then further incubated with IL-4 (50 ng/mL), for 24 h. Eotaxin-1 production was analyzed by ELISA; (**b**) Cell viability was measured, using the MTT assay. Data are representative of at least three independent experiments. Results are mean ± standard deviation (SD) (*n* = 3).¶ *p* < 0.05 *vs.* IL-4-untreated control. ** *p* < 0.05 *vs.* IL-4-treated control.

An eotaxin-1 ELISA was performed, using signaling inhibitors (JAK1, STAT6) to demonstrate the involvement of JAK/STAT signaling in eotaxin-1 expression. As shown in Figure 5a, IL-4-induced eotaxin-1 expression decreased, following treatment with JAK1 inhibitor, pyridone 6 and STAT6 inhibitor, AS1517499 respectively. Additionally, we found that these results were not attributed to signal inhibitors cytotoxicity (Figure 5b).

### 2.6. Eotaxin-1 Induced Eosinophil Recruitment was Inhibited by Synephrine

**Figure 6:**
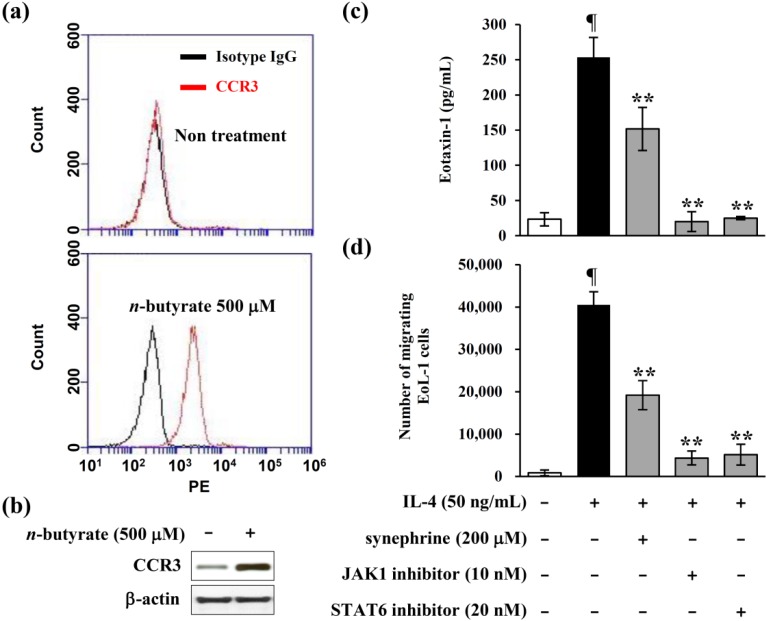
Effects of synephrine on the recruitment of eoinophils. EoL-1 cells were incubated for 7 days at 37 °C, in RPMI medium containing 500 μM *n*-butyrate. (**a**) The cell surface expression of CCR3 was determined by flowcytometry. Red histograms show CCR3, and isotype controls are represented in the black histograms; (**b**) Cell lysates were prepared at 7 days, and then subjected to Western blot analysis. The bands for CCR3 were detected, and normalized to that of β-actin. Migration assays were performed, using CytoSelect 24-well Cell Migration Assay kits. Human normal fibroblasts, 5 × 10^4^ cells were seeded in lower chambers, and incubated for 24 h. Lower chambers were pretreated with synephrine, JAK1 inhibitor, pyridine 6 and STAT6 inhibitor, AS1517499 respectively, and then stimulated with IL-4. Differentiated EoL-1 cells, 2 × 10^6^ cells were added to the upper chamber of the transwell plates, with 3 μM pore-size filters. IL-4-induced eotaxin-1 was measured using ELISA, in lower chambers (**c**). The number of eosinophils migrating from upper chambers to lower chambers was determined by fluorescence (**d**). Data are representative of at least three independent experiments. Results are mean ± standard deviation (SD) (*n* = 2). ¶ *p* < 0.05 *vs.* IL-4-untreated control. ** *p* < 0.05 *vs.* IL-4-treated control.

EoL-1 cells, a recently established human eosinophilic leukemia cell line, have cytological features of myeloblasts under normal culture conditions; and differentiate not only phenotypically, but also functionally into eosinophils, by a number of stimuli [26]. Previous study has reported that *n*-butyrate induces the differentiation of eosinophilic leukemia EoL-1 cells into mature eosinophils [27]. The activities of eotaxin-1 are mediated by binding to the chemokine receptor CCR3 that is expressed on eosinophils [28]. To investigate the effect of *n*-buytrate on the differentiation of EoL-1 cells into eosinophils, we treated EoL-1 cells with *n*-butyrate 500 μM for 7 days. Flow cytometry and western blot analysis were performed, to measure the differentiation of EoL-1 cells into eosinophils. As shown in Figure 6a, *n*-butyrate induced the expression of CCR3, cell surface marker of eosinophils on EoL-1 cells. In addition, *n*-butyrate induced the total CCR3 protein level on EoL-1 cells (Figure 6b). Finally, differentiated EoL-1 cells were used, for further experiment of eosinophil recruitment assay. Eotaxin-1 is a potent chemoattractant for eosinophils, and a critical mediator, during the development of eosinophilic inflammation. Thus, Transwell migration assay system using normal human fibroblasts (NHFs) and differentiated EoL-1 cells was introduced, to confirm this result. In this system, NHFs and differentiated EoL-1 cells were co-cultured in a two-compartment Transwell system. 

NHFs in the lower chamber were pretreated with synephrine and signal inhibitors (JAK1 inhibitor, and STAT6 inhibitor), and then stimulated with IL-4. As shown in Figure 6c, IL-4-stimulated NHFs produced eotaxin-1, which is inhibited by synephrine and signal inhibitors. IL-4-induced eotaxin-1 recruited differentiated EoL-1 cells to the lower chamber, at high concentration of eotaxin-1 compartment in the NHFs/differentiated EoL-1 cells co-culture system (Figure 6d). Also, the eotaxin-1-mediated EoL-1 cells transmigration into the lower chamber was attenuated by synephrine treatment (Figure 6d). In addition, JAK1 and STAT6 inhibitors also inhibited transmigration of differentiated EoL-1 cells into the lower chamber, by inhibiting JAK1 and STAT6 activation, for the expression of eotaxin-1 induced by IL-4 (Figure 6d). These results indicated that synephrine attenuated the eosinophil transmigration, through inhibition of the eotaxin-1 production.

## 3. Experimental Section

### 3.1. Cell Culture and Materials

NIH/3T3 mouse fibroblast cell line and Normal human fibroblasts (NHFs) were maintained in Dulbecco’s Modified Eagle’s Medium (DMEM, Gibco, Carlsbad, CA, USA), containing 10% Fetal bovine serum (FBS, Gibco) and 1% penicillin/streptomycin (Invitrogen, Carlsbad, CA, USA), at 37 °C, under 5% CO_2_. The EoL-1 human eosinophilic leukemia cell line was obtained from the European Collection of Cell Cultures (ECACC, Salisbury, UK). EoL-1 cells were cultured in RPMI1640 containing 10% FBS and 1% penicillin/streptomycin, at 37 °C, under 5% CO_2_.

### 3.2. High Performance Liquid Chromatography

A Waters (Milford, MA, USA) HPLC system with a 600 Controller, 996 Photodiode Array Detector, 616 Quaternary pump, and a 717 Autosampler was used for analysis of synephrine. Data acquisition was achieved using the Waters Empower software. All chromatographic separations were conducted on a Luna^®^ 5 μM C18 column (150 mm × 4.6 mm, 5 μm, Phenomenex, Torrance, CA, USA) at ambient temperature with detection at 273 nm. The mobile phase consisted of 0.1% trifluoroacetic acid in water and acetonitrile (97:3, v/v) for 15 min.

### 3.3. Cytokines and Pharmacological Inhibitors

Synephrine was purchased from Sigma Aldrich (St. Louis, MO, USA). Recombinant mouse IL-4, human IL-4, human eotaxin-1, human CCR3-PE, and mouse IgG2b κ isotype PE were all purchased from eBioscience (San Diego, CA, USA). Pyridone 6 (JAK Inhibitor I) was purchased from Calbiochem (Darmstadt, Germany). Phospho-STAT6 and total-STAT6 antibody were purchased from Abcam (Cambridge, UK), and STAT6 inhibitor was purchased from Axon Medchem (Groningen, NL, USA). Phospho-JAK1 and β-actin antibody were purchased from Cell Signaling Technology, Inc. (Beverly, MA, USA).

### 3.4. Cell Viability Assay

Cell viability was measured using the MTT (3-[4, 5-dimethylthiazol-2-yl]-2,5-diphenyltetrazolium bromide; USB Corp., Cleveland, OH, USA) assay. Cells were plated in triplicate wells of 12-well plates, and incubated overnight. The cells were then treated with synephrine for 24 h, under a serum-free condition. Then, MTT reagent (1 mg/mL) was added to each well, and the cells were incubated for 3 h. The medium was removed, and the cells were solubilized with dimethyl sulfoxide (DMSO, Sigma, St. Louis, MO, USA). The absorbance was measured by spectrophotometer at a wavelength of 570 nm.

### 3.5. Enzyme-Linked Immunosorbent Assay (ELISA)

Eotaxin-1 concentrations were quantified in cell culture supernatants, after treatment with synephrine induced by IL-4, using a commercially available ELISA kit (eBioscience). Cell culture supernatants were collected 24 h after treatment with 200 μM synephrine, and assayed for eotaxin-1. The standard curve was linearized, and subjected to regression analysis. Eotaxin-1 concentrations were determined, using a standard curve. All samples and standards were measured in duplicate.

### 3.6. Transient Transfection and Luciferase Assay

NIH/3T3 cells were transfected with the eotaxin-1 luciferase reporters, using the SuperFect^®^Transfection Reagent (Qiagen, Hilden, Germany). After 24 h incubation, cells were incubated in the presence or absence of synephrine induced by IL-4, for 24 h. The cells were then harvested and lysed, and supernatants were assayed for luciferase activity. Luciferase activity was determined, using a Dual Luciferase Assay System (Promega, Madison, WI, USA), and an Infinite^®^ 200 PRO luminometer (Tecan, AG, Männedorf, Switzerland). Activity was expressed as the ratio of STAT6-dependent firefly luciferase activity, to control thymidine kinase Renilla luciferase activity.

### 3.7. Immunohistochemistry

STAT6 translocation was determined by immunohistochemistry. An aliquot of 1 × 10^2^ cells was seeded in 96-well plates, cultured for 24 h, and then treated with IL-4 in the presence or absence of synephrine, for 60 min. The cells were fixed in 4% paraformaldehyde, washed with PBS, permeabilized with 0.2% Triton X-100, and incubated with a rabbit polyclonal antibody to STAT6 (1:200, 5% goat serum in PBS) for 2 h, followed by fluorescein isothiocyanate (Santa Cruz Biotechnology, Santa Cruz, CA, USA) goat anti-rabbit IgG (1:200), for 1 h. After three washes with PBS, the cells were incubated with 0.5 μg/mL Hoechst 33342 (Invitrogen), for 5 min. The cells were then examined, using the IN Cell Analyzer 1000 (GE Healthcare Lifescience, Uppsala, Sweden).

### 3.8. Total RNA Extraction, cDNA Synthesis, and Quantitative PCR

Total RNA extraction was carried out using the RNeasy kit (Qiagen), according to the manufacturer’s instructions. The first cDNA was synthesized using a PrimeScript First Strand cDNA Synthesis kit (Takara Bio, Japan), according to the manufacturer’s instructions. Eotaxin-1 mRNA expression was measured by real-time quantitative PCR, using a specific TaqMan probe (CCL11, Applied Biosystems, Foster City, CA, USA). TaqMan gene expression assays were performed on the ABI PRISM 7300 System (Applied Biosystems), according to the manufacturer’s instructions. All samples were analyzed in triplicate, and the levels of the detected mRNAs were normalized to control mouse beta-2-microglobulin (Applied Biosystems) values. The normalized data were used to quantify the relative levels of a given mRNA, according to *Δ*Ct analysis.

### 3.9. Cell Migration Assay

We used the Cytoselect Cell Migration Assay kit (Cell Biolabs, San Diego, CA, USA), according to the manufacturer’s instructions. Briefly, differentiated eosinophilic EoL-1 cells and human fibroblasts were co-cultured in two-compartment Transwell system. For eosinophilic EoL-1 cells, 2 × 10^6^ cells in 400 μL serum free medium were added to the upper chamber of the Transwell plates, with 3-μm-pore filters. Lower chambers were first seeded with fibroblasts at 5 × 10^4^ cells in 500 μL serum free medium, for 24 h. Synephrine and/or IL-4 were added to the lower wells, for 24 h. After 24 h incubation, migratory EoL-1 cells were subsequently lysed, and detected by fluorescence measurement.

### 3.10. Statistical Analysis

All data are expressed as means ± standard deviations. Differences between the control and the treatment group were evaluated by one way ANOVA. *p* < 0.05 was considered statistically significant. 

## 4. Conclusions

Our results show that synephrine regulates IL-4-induced eotaxin-1 expression, by inhibiting STAT6 phosphorylation. In spite of the importance of these factors on the therapeutic target of allergic inflammation, only a few STAT6 inhibitors have been disclosed, to date. Therefore, STAT6 inhibitory agents might be useful for the treatment of allergic inflammation, as a target for therapeutic intervention. In this study, we propose that the inhibitory effects of synephrine on eotaxin-1 expression may be at least partially due to defective eotaxin-1 production, in allergic inflammation and atopic dermatitis. 
